# Photocatalytic degradation of organic dyes using reduced graphene oxide (rGO)

**DOI:** 10.1038/s41598-024-53626-8

**Published:** 2024-02-13

**Authors:** Mizaj Shabil Sha, Hayarunnisa Anwar, Farzana N. Musthafa, Hamad Al-Lohedan, Sarya Alfarwati, Jothi Ramalingam Rajabathar, Johaina Khalid Alahmad, John-John Cabibihan, Muthusamy Karnan, Kishor Kumar Sadasivuni

**Affiliations:** 1 Center for Advanced Materials, Qatar University, PO Box 2713, Doha, Qatar; 2 Chemistry Department, College of Science, King Saud University, P.O. Box. 2455, Riyadh, 11451 Kingdom of Saudi Arabia; 3 Department of Mechanical and Industrial Engineering, College of Engineering, Qatar University, P.O. Box. 2713, Doha, Qatar; 4 Grassland and Forage Division, National Institute of Animal Science, Rural Development Administration, Wanju, South Korea

**Keywords:** Biochemistry, Environmental sciences

## Abstract

Photocatalysts have developed into a successful strategy for degrading synthetic and organic toxins, such as chemicals and dyes, in wastewater. In this study, graphene oxide was reduced at different temperatures and used for degrading indigo carmine and neutral red dyes. The wide surface areas, strong adsorption sites, and oxygen functionalities of reduced graphene oxide (rGO) at 250 °C (rGO-250) produced more photocatalytic degradation efficiency and adsorption percentage. The catalyst dosage, initial dye concentration, solution pH and recyclability were all used to optimize the photocatalytic activity of rGO-250. This research presents a capable nano-adsorbent photocatalyst for the efficient degradation of organic dyes. GO and rGOs were also investigated for carbon dioxide (CO_2_) absorption properties. Results showed that rGO-250 has better CO_2_ adsorption properties than other rGOs. Overall, it was observed that rGO-250 has better photocatalytic and CO_2_ adsorption capabilities compared to graphene oxide reduced at different temperatures.

## Introduction

Different facets of the textile manufacturing sectors depend heavily on dyes. However, because the dyes cannot degrade, they are frequently dumped carelessly into the aqueous environment^1,2^. Most dyes contain poisonous and carcinogenic characteristics that could harm ecosystems and people’s health. Due to their complex aromatic structures, dyes resist natural processes that cause degradation^3,4^. Because of this, various treatment methods have been used to remove dyes. Due to the availability of low-cost adsorbents with significant adsorption capacities, the adsorption method is the most popular strategy utilised in the industry^5^. Due to its simplicity, photocatalysis is yet another widely utilised technology. Reactive oxygen species (ROS) are generally acknowledged to include both superoxide (·O2^−^) and hydroxyl (·OH^−^) radicals in the photooxidation of dye pollution. The amount of surface area of the materials utilised significantly impacts the removal of dyes in both reactions^6,7^.

Reduced graphene oxide (rGO), a chemically changed graphene, is more economically suited for mass production than pristine graphene. rGO frequently finds use in the production of composites based on graphene^8^. Various techniques, including microwave, thermal, photo-thermal, chemical, photo-chemical, and microbial/bacterial, can create rGO^9^. The chemical technique seems advantageous due to its low cost, ease of use, and large output. In particular, there are three key processes in this method's preparation of rGO^10^. The first stage entails the oxidation of graphite to produce graphite oxide, which introduces surface oxygen functions to the graphene layers^11^. Because graphite oxide has oxygenated surface functions such as carboxyls, hydroxyls, and epoxides, it may disperse in polar solvents and form stable dispersions^12^. Then, graphite oxide is exfoliated using mechanical stirring or sonication to produce graphene oxide (GO) with one or a few layers. Finally, by removing the surcial oxygen functions, GO is converted to rGO^11^. Adsorption reactions to remove organic and inorganic pollutants have historically used carbon-based compounds. The most often used adsorbent is activated carbon. Recently, rGO utilisation in dye adsorption applications has increased^13–15^. Because of some flaws in the graphitic domains and the remaining surficial oxygen functions, rGO is considered effective for the adsorptive removal of dyes. In general, rGO interacts with dyes by structural conjugation, hydrophobic association, electrostatic interaction, and π–π interaction^16,17^. Several different dyes can adsorb on rGO thanks to these interactions. It is ideal to have rGO with a wide surface area and high porosity to increase the dye adsorption capacity, and this is possible by managing the quality of the GO precursor and the reduction process^18,19^.

The technological advantages of photocatalysis are currently manifested through the photocatalytic degradation of dyes. Typically, electrons are stimulated, forming electrons and holes, when the energy of the light absorbed is equal to or less than the band gap of the photocatalyst. The photogenerated electron–hole pairs then use the nearby oxygen and water molecules to make ROS, which destroy the dye molecules^20,21^. Numerous studies have described using rGO in the photocatalytic removal of dye contaminants. The novelty of using reduced graphene oxide (rGO) for dye degradation lies in its remarkable catalytic properties and potential to revolutionize wastewater treatment methods. Unlike traditional approaches, rGO-based catalysts offer several unique advantages:

1. Enhanced catalytic activity: rGO exhibits high surface area and excellent electron transfer properties, enabling it to catalyze the degradation of dyes and other organic pollutants efficiently.
2. Improved reaction kinetics: Using rGO can significantly accelerate the degradation kinetics of dyes, reducing the time required for wastewater treatment.
3. Sustainability: rGO can be synthesized from abundant and inexpensive graphite sources, making it a sustainable and cost-effective option for environmental remediation.
4. Versatility: rGO can be functionalized and tailored to target specific dye molecules, increasing its versatility for various wastewater treatment applications.
5. Low environmental impact: Compared to some traditional chemical catalysts, rGO-based systems are often less harmful to the environment, aligning with the growing emphasis on green and sustainable technologies.

In summary, using rGO as a catalyst for dye degradation represents a novel and promising approach that addresses both the efficiency and sustainability challenges associated with wastewater treatment. This innovation has the potential to contribute significantly to cleaner water resources and reduced environmental pollution.

In this study, rGO was produced using a straightforward, eco-friendly solvothermal method without any harmful reducing chemicals, and it demonstrated outstanding adsorption capabilities and photoactivity towards the removal of IC and NR dye. The investigation examined the optimisation of extraneous elements such as catalyst concentration, initial dye concentration, light intensity, and pH.

## Experiment

### Chemicals

All the chemicals used (graphite powder, potassium permanganate (KMnO_4_), sulphuric acid (H_2_SO_4_, 98%), hydrogen peroxide (H_2_O_2_, 30%) and ethanol (C_2_H_5_OH) were of analytical grade, procured from Sigma Aldrich. Deionized water was used as the solvent for preparing practical solutions.

### Methodology

#### Synthesis of GO

Graphite powder pretreatment is initially carried out to prepare Graphene oxide (GO). In the beginning, graphite (1 g) was added to H_2_SO_4_ (25 ml). It was stirred in an ice bath for uniform suspension for a few minutes. After 20 min, the ice bath was removed.

A modified Hummer method is used to prepare GO from graphene. KMnO_4_ (3 g) was added slowly to the solution and stirred for 3 h. 50 ml of distilled water was added slowly into the solution after 3 h. 50 ml of H_2_O_2_ was added to cease the process. When a brown colour is observed, add 100 ml of distilled water. Add 5 ml of H_2_O_2_ and centrifuge the mixture. Wash with H_2_O to collect the GO. For drying, it was placed in an oven at 60 °C for 2 h.

#### Preparation of reduced Graphene oxide (rGO) at different temperatures

To reduce GO to rGO, the solvothermal method was used. The mixture is then heated at different temperatures for the reduction process. Initially, 20 ml of C_2_H_5_OH and 200 mg of GO were mixed with 10 ml of deionized water. After vigorously sonicating the mixture for 30 min, a stable GO dispersion was produced. After that, the mixture was heated for 2 h at 100, 150, 200, and 250 °C in an 80 cc stainless steel autoclave lined with Teflon. The samples were designated rGO-x, with x standing for the investigated reduction temperatures. The rGO samples were subsequently filtered, washed, and dried.

#### Characterization of reduced graphene oxide

The X-ray diffractometer, a Malvern Panalytical Xpert (45 kV, 40 mA), was utilised to collect the XRD pattern from a Cu target. The materials were dispersed in ethanol before being put onto the copper grid for transmission electron microscopy (TEM) with an FEI Tecnai G2 S-Twin FEG 200 kV TEM. The sample was prepared for transmission electron microscopy (TEM) by dissolving the powder in isopropanol using a bath sonicator for 10–20 min. After being drop cast onto 200-grit carbon film, the sample was examined. An FTIR spectrometer called the Thermo Nicolet Nexus 670 was used to assess the purity of nanopowder. A Biochrom UV–Vis Spectrophotometer with a 190–1100 nm scanning range was used for the characterisation. A 300–750 nm scanning range and a medium scan speed were used in this case. The surface areas, pore diameters, and pore volumes of GO and rGOs were examined using Brunauer–Emmett–Teller (BET) analysis using the Micromeritics ASAP 2020 Surface Area and Porosity Analyzer (Georgia, USA). Before measurement, the samples were degassed at 150 °C for 24 h to eliminate moisture.

#### Dye degradation analysis

To 20 ml of distilled water, 15 mg of the photocatalyst (GO&rGO' s) were measured and added. The fluid was sonicated for around 30 min to produce a consistent suspension. Approximately 5 ml of this solution is added to the 40 ml of NR and IC aqueous solutions. The sample solution was left in the dark for roughly 30 min, allowing the reaction to reach adsorption equilibrium. After being maintained in the dark for 30 min, three millilitres of the sample are removed and subjected to UV–vis spectroscopy examination. Magnetic stirring was used to keep the solution stable while it was in the sun. A sample of the supernatant solution was taken every 20 min until three hours into the procedure. At this point, it was examined with UV–vis technology. The absorbance and wavelength were plotted. It was also investigated how varied catalyst loadings (1, 2, 3, 4, and 5 mg) of GO and rGOs, pH effect (3, 7, 10), recyclability, and initial dye concentrations (25, 40, 50, and 75 ppm) of two dyes would affect the process^22^.

The following equations were used to calculate the photodegradation efficiency (% Cdeg) and adsorption percentage (% Cads):

1 $$\% \,{\text{C}}_{{{\mathrm{ads}}}} = \left[ {\left( {{\text{C}}_{{{\mathrm{initial}}}} - {\text{C}}_{0} } \right)/{\mathrm{C}}_{{{\text{initial}}}} } \right]*100\%$$

2 $$\% \,{\text{C}}_{{{\mathrm{deg}}}} = \left[ {\left( {{\text{C}}_{0} - {\text{C}}_{{\mathrm{t}}} } \right)/{\text{C}}_{0} } \right]*100\% ,\quad 0 \le {\text{t}} \le 3$$

The dye concentrations at the start, after attaining adsorption–desorption equilibrium, and at time t are designated as C_initial_, C_0_, and C_t_, respectively. The pseudo-first-order kinetic equation shown below was then used to simulate the photocatalytic degradation reaction:

3 $$- {\text{ln }}\left( {{\text{C}}_{{\mathrm{t}}} /{\text{C}}_{0} } \right) = {\text{kt}};\quad 0 \le {\text{t}} \le 3$$

where k (time^−1^) is the rate constant for pseudo-first-order.

#### Evaluation of CO_2_ adsorption properties

To evaluate the CO_2_ adsorption properties, we dissolved 50 mg of GO and rGOs in 10 ml water. After passing CO_2_ for 15 s in all the samples, it is titrated against 0.1 N NaOH, adding 10 drops of indicator. Noted the colour change and volume of NaOH for calculating the amount of CO_2_ absorbed using the following equations.

4 $${\text{N}}_{1} {\text{V}}_{1} = {\text{N}}_{2} {\text{V}}_{2}$$

5 $${\text{N}}_{1} = \left( {{\text{N}}_{2} {\text{V}}_{2} } \right)/{\text{V}}_{1}$$

where V_1_—Volume of the sample used; N_1_—Normality of the sample; V_2_—Volume of NaOH; N_2_—Normality of NaOH

The amount of CO_2_ adsorbed was calculated using the following equation,

6 $${\text{Amount of CO}}_{2} = {\text{Equivalent weight of CO}}_{2} *{\text{N}}_{1}$$

## Results and discussion

### Characterization

**Figure 1:**
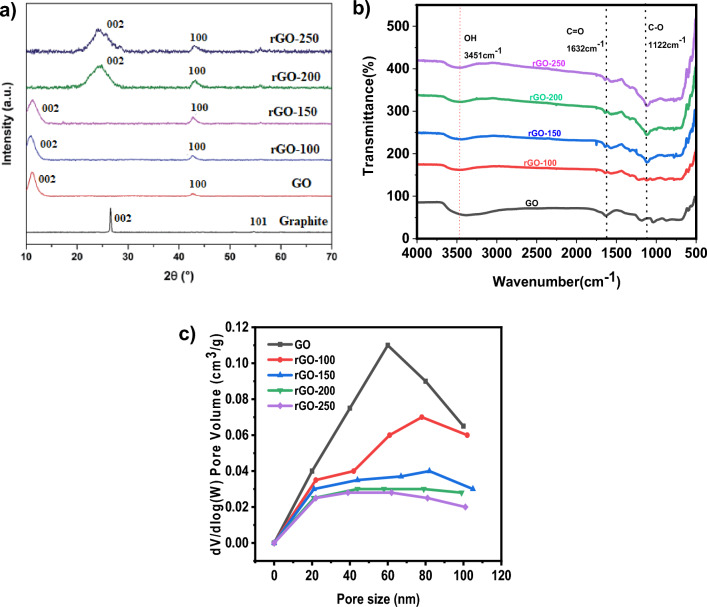
(**a**) XRD patterns and (**b**) FTIR spectra of graphite, GO, rGO-100, rGO-150, rGO-200 and rGO-250 (**c**) Pore size distributions of GO,rGO-100, rGO-150, rGO-200 and rGO-250.

In Fig. 1a, at 2 = 26.570° and 2 = 54.700°, respectively, graphite showed an intense (002) peak and a very modest (101) peak. Both peaks were associated with JCPDS card 41-148713 and supported graphite structure. After oxidation, the (002) peak moved to 2 = 11.030°, caused by incorporating oxygenated functionalities and water molecules into the graphene layers. Despite this, the (002) peak of GO was still discernible during reduction at 100 and 150 °C, maybe due to the low reduction temperatures that only slightly converted GO to rGO. The (002) peak of GO started to fade as the reduction temperature rose, but a new, broader (002) peak of rGO started to appear at 2 = 24.890° for rGO-200 and 2 = 23.850° for rGO-250. The shifting of the (002) peak indicated that the *sp*^2^ carbon structure had recovered partially^23^. Also, the (100) GO peak at 2θ = 42.630° was detected in all rGOs, resembling the turbostratic band of disordered carbon materials.

Figure 1b displays the FTIR spectra of rGO samples produced at various reaction temperatures. The GO surface contains a large number of functional groups. Figure 1b shows the peaks of C–O (alkoxy) stretch, O–H (hydroxyl) vibration, C=C aromatic stretch vibration, and C=O stretch on rGO surfaces at 1122, 1632, and 3451 cm^−1^. As the temperature rose, so did the peak intensity corresponding to the C=O vibration band at 1632 cm^−1^. From 100 to 250 °C, there was a decrease in the hydroxyl group's (3451 cm^−1^) permeability intensity^15^. As the temperature rises, the absorbed water molecules intercalate and vaporise. Furthermore, the hydrophilic characteristics of GO are lost, as well as the presence of functional groups containing oxygen^24^.

Figure 1c shows the pore size distribution curves for GO and rGOs. According to Fig. 1c, the total pore volume decreased from 0.11 to 0.037 cm^3^ g^−1^ when the GO was lowered from 100 to 150 °C, decreasing the surface area (Table 1). This resulted most likely from the GO's aggregation action following the decrease. The considerable loss of oxygen functional groups led to the production of greater pore volume, which in turn resulted in a pronounced increase in surface area for rGO-250 at higher reduction temperatures^25^.

**Table 1:** BET textural parameters of rGOs.

Sample	Surface area (m^2^ g^−1^)	Pore size (nm)	Pore volume (cm^3^ g^−1^)
rGO-100	1.85	59.42	0.11
rGO-150	0.57	64.19	0.037
rGO-200	0.415	72.18	0.03
rGO-2500	3.164	56.89	0.18

**Figure 2:**
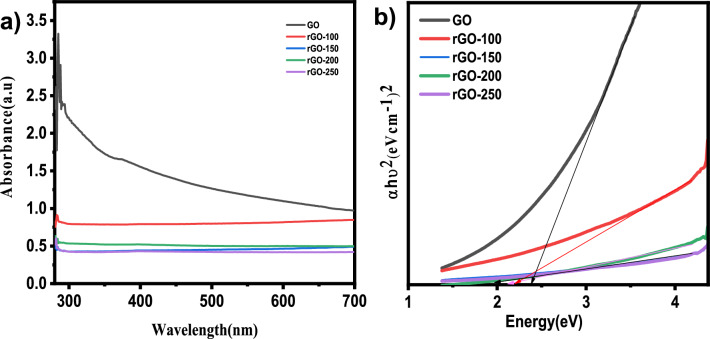
(**a**) UV–vis absorption spectra and (**b**) Tauc plots of graphite, GO, rGO-100, rGO-150, rGO-200 and rGO-250.

The UV–vis absorption spectra of GO and rGOs are shown in Fig. 2a. The aromatic C=C bond π–π* transition of GO results in an absorption peak at 284 nm. The temperature ranged from 100 to 250 °C, and as a result, the peak location gradually shifted to lower wavelengths^13^. Peak shifting partially restored the sp^2^ conjugation, leading to a narrower band gap^17^. The optical band gaps of GO and rGOs were subsequently determined using the linear fits of the Tauc plots, as seen in Fig. 2b. Table 2 shows the band gap of the samples. The band gap of GO was 2.80 eV, which was in line with results from earlier literature^26^.

**Table 2:** Bandgap of components.

Sl. no.	Compound	Bandgap (eV)
1	GO	2.8
2	rGO-100	2.4
3	rGO-150	2.2
4	rGO-200	2.1
5	rGO-250	2

**Figure 3:**
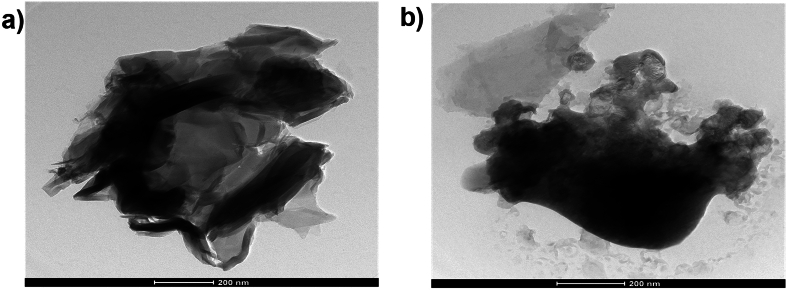
TEM image of (**a**) GO and (**b**) rGO-250.

The TEM micrographs in Fig. 3a and b show the sheet-like morphology of GO and wrinkled rGO caused by functional group evaporation and thermal instability of GO during annealing^19^. The GO displayed a pristine surface and a closely packed lamellar and plate structure consistent with previously made GO. The distinctive sheet-like silky waves, wrinkled appearance, and clumped structure of rGO, in contrast to GO, were between 20 and 100 nm in size^27^.

### Photocatalytic activity of rGOs

**Figure 4:**
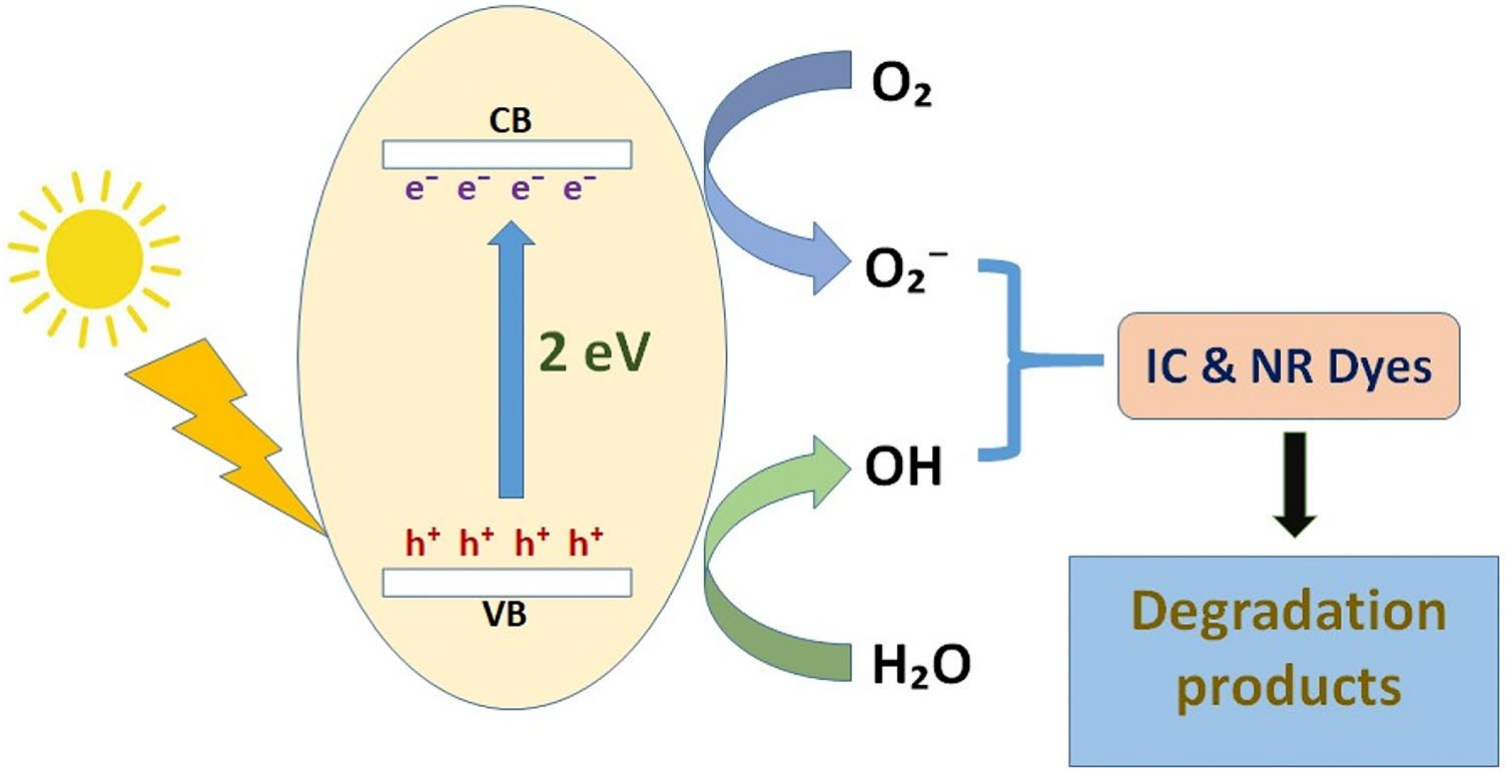
Mechanism of dye degradation.

Structural conjugation, hydrophobic association, electrostatic interaction, and -interaction is how rGO interacts with dyes. These interactions allow a variety of dyes to adsorb on rGO. To maximise the dye adsorption capacity, it is essential to have rGO with a large surface area and high porosity. This is achievable by carefully controlling the GO precursor's quality and the reduction process. The mechanism of dye degradation involves the excitation of dye from its ground state (Dye) to its triplet excited state (Dye*) under visible light photon (λ > 400 nm). An electron injection into the conduction band transforms this excited state dye species into a semi-oxidized radical cation (Dye^+·^). Superoxide radical anions (O_2_^-·^) are produced as a result of an interaction between these trapped electrons and the dissolved oxygen in the system, which in turn causes the creation of hydroxyl radicals (OH^·^) (Fig. 4).

**Figure 5:**
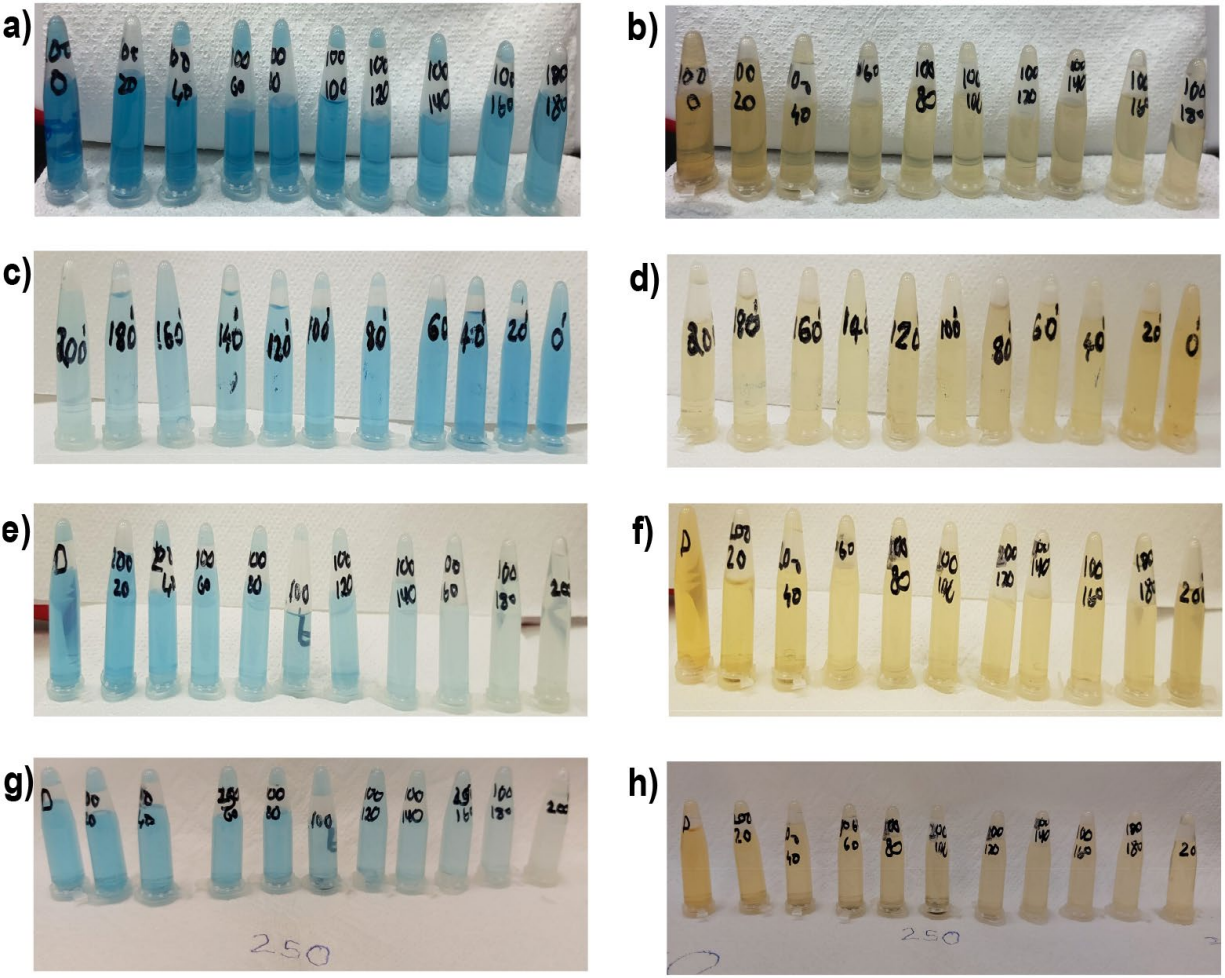
Dye degradation analysis of (**a**,**b**) rGO-100 in IC and NR respectively (**c**,**d**) rGO-150 in IC and NR respectively (**e**,**f**) rGO-200 in IC and NR respectively (**a**,**b**) rGO-250 in IC and NR respectively.

**Figure 6:**
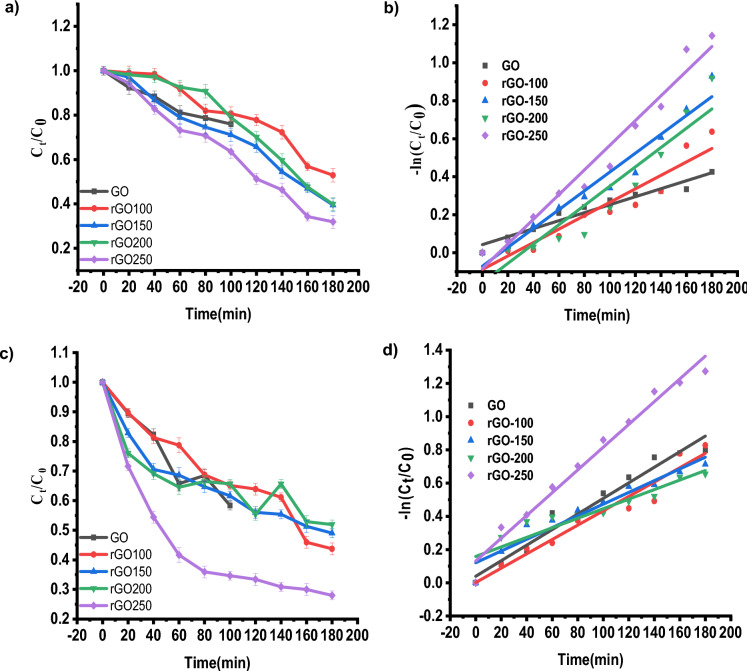
(**a**,**c**) Degradation of IC and NR dyes normalized against adsorption, (**b**,**d**) Pseudo-first-order kinetic plot of IC and NR dyes, respectively, in the presence of GO, rGO-100, rGO-150, rGO-200 and rGO-250.

Figure 5 illustrates the photocatalytic activity of different rGOs in IC and NR, respectively. The solution is collected after the dyes have been exposed to the sun for a predetermined time. It is measured how much UV–vis is absorbed (Figure S1, S2, S3 and S4). Table 3 shows the concentration–time profile (C_t_/C_0_) and degradation efficiency (%), which were derived using the absorbance values obtained for the initial dye concentration (C_0_) and the absorbance values acquired at certain time intervals (C_t_). The photodegradation of dyes was found to be much more effective with rGO-250.It may be attributed to the fact that the rGO-250 may have the large and optimum surface area for the highest adsorption of dyes^28,29^. Figure 6a and c depict GO and rGO photoactivity, respectively, while Fig. 6b and d depict the pseudo-first-order fitting of photocatalytic dye degradation. Table 3 shows the values for % C_ads_, % C_deg_, k, and R^2^. rGO-100, rGO-150, and rGO-200 behaved similarly to GO in the dye adsorption process to other rGOs because low reduction temperatures were insufficient for GO reduction. Because of its large surface area, rGO-250 had the highest dye adsorption^30^, with an adsorption percentage of 29.26 for IC and 25.23 for NR, respectively. The photocatalytic degradation efficiency of rGO-250 was 36.28 and 25.73 for IC and NR, respectively.

**Table 3:** Effect of reaction temperature on adsorption percentage, photocatalytic degradation efficiency, and photocatalytic degradation rate of IC and NR dye.

Sample	Adsorption percentage (%)		Photocatalytic degradation efficiency		k (min^−1^)		R^2^	
	IC	NR	IC	NR	IC	NR	IC	NR
GO	10.4	9.8	8.23	9.1	0.0021	0.00469	0.95356	0.96415
rGO-100	11.5	12.4	14.14	12.37	0.00354	0.00432	0.90567	0.95259
rGO-150	17.77	16.53	19.76	15.48	0.00496	0.00453	0.95599	0.9337
rGO-200	23.2	20.35	21.1	19.86	0.0050	0.00487	0.9017	0.95632
rGO-250	29.26	25.23	36.28	25.73	0.0065	0.00685	0.96642	0.97612

The statistical analysis of linear curve fit was performed using origin software. This statistical analysis aimed to regulate factors affecting dye photodegradation performance. The statistical analysis results of fitted curves for indigo carmine and neutral dye are represented in Table 4. Statistical evidence, such as Fisher variation ratio (F-value) and probability value (*P* value), was used to assess the linear fit for estimating photodegradation performance. Additionally, degrees of freedom (DF), mean squares (MS), and sum of squares (SS) were calculated. The F-value of 14.55 indicates the model is significant. Noise has a 0.01% probability of causing a ''Model F-value". Significant model terms are indicated by “'Probability > F” values < 0.0500.

**Table 4:** Statistical parameters of indigo carmine and neutral red dyes.

Dyes	Sample		DF	MS	SS	F value	Probability > F
Indigo carmine	GO	Model	1	0.14595	0.14595	164.26316	1.29622E−6
		Error	8	8.88523E−4	0.00711		
		Total	9		0.15306		
	rGO-100	Model	1	0.41312	0.41312	76.80988	2.25172E−5
		Error	8	0.00538	0.04303		
		Total	9		0.45615		
	rGO-150	Model	1	0.81186	0.81186	173.77495	1.04442E−6
		Error	8	0.00467	0.03738		
		Total	9		0.84923		
	rGO-200	Model	1	0.85215	0.85215	58.76066	5.93053E−5
		Error	8	0.0145	0.11602		
		Total	9		0.96816		
	rGO-250	Model	1	1.39342	1.39342	230.2529	3.52357E−7
		Error	8	0.00605	0.04841		
		Total	9	0.14595	1.44183		
Neutral red	GO	Model	1	0.14595	0.14595	164.26316	1.29622E−6
		Error	8	8.88523E−4	0.00711		
		Total	9		0.15306		
	rGO-100	Model	1	0.41312	0.41312	76.80988	2.25172E−5
		Error	8	0.00538	0.04303		
		Total	9		0.45615		
	rGO-150	Model	1	0.81186	0.81186	173.77495	1.04442E−6
		Error	8	0.00467	0.03738		
		Total	9		0.84923		
	rGO-200	Model	1	0.85215	0.85215	58.76066	5.93053E−5
		Error	8	0.0145	0.11602		
		Total	9		0.96816		
	rGO-250	Model	1	1.39342	1.39342	230.2529	3.52357E−7
		Error	8	0.00605	0.04841		
		Total	9	0.14595	1.44183		

#### Effect of pH

**Figure 7:**
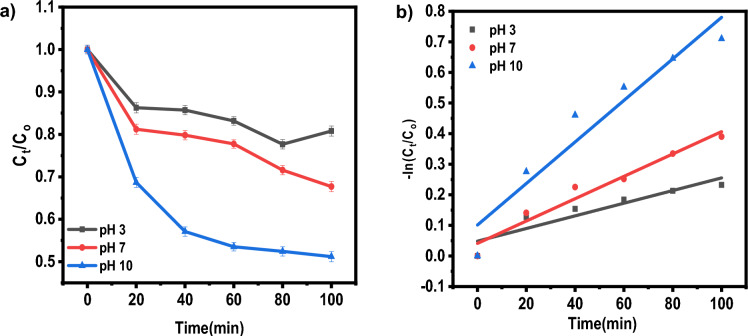
(**a**) Effect of pH on photocatalytic degradation of NR normalized under adsorption. (**b**) The plot of the log of concentration with time.

15 mg of rGO-250 in 20 ml distilled water kept for sonication of about 30 min. Two sets of dye solutions (IC&NR) were made of pH 3, 7 and 10. 5 ml from the catalyst solution was added to 40 ml of aqueous solutions of dyes of 25 ppm of concentrations (Fig. 7). The degradation (91.85% for IC and 90.17% for NR) is much more pronounced in the basic medium (pH 10) for both dyes than in the acidic (pH 3, 85.15% for IC and 84.58%for NR) and neutral medium (87.92% for IC and 86.47% for NR) (Table 5). This may be because the solution contained more hydrogen ions under acidic conditions. Many hydrogen ions competed with the dye molecules for unoccupied adsorption sites on the catalyst surface. In this case, IC is an anionic dye, and NR is a neutral dye^28^.

**Table 5:** Effect of pH on adsorption percentage, photocatalytic degradation efficiency, and photocatalytic degradation rate of IC and NR dye.

pH of sample	Adsorption percentage (%)		Photocatalytic degradation efficiency		k (min^−1^)		R^2^	
	IC	NR	IC	NR	IC	NR	IC	NR
pH 3	80.58	76.82	85.15	84.58	0.0041	0.00207	0.937	0.857
pH 7	81.35	78.14	87.92	86.47	0.0055	0.00365	0.956	0.957
pH 10	82.92	81.69	91.85	90.17	0.0072	0.00679	0.989	0.924

#### Effect of initial dye concentrations

**Figure 8:**
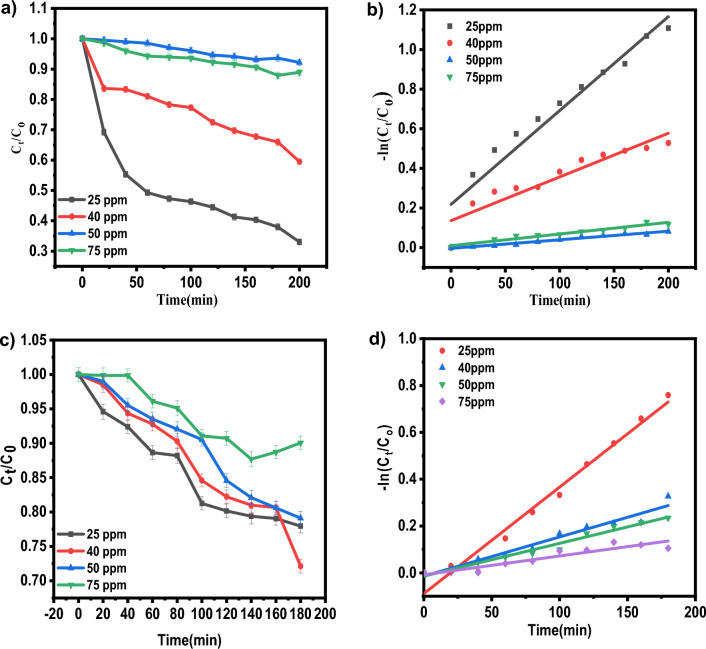
(**a**,**c**) Effect of initial dye concentration of IC, NR on photocatalytic degradation of dyes normalized against adsorption. (**c**,**d**) The plot of the log of concentration with time.

The photocatalytic degradation of dyes was studied using various initial concentrations such as 25, 40, 50 and 75 ppm.15 mg of catalyst in 20 ml of DI water was added to 40 ml of each dye (IC, NR) of varied initial concentration (Table 6). An initial concentration of 25 ppm was observed to have greater dye degradation efficiency than other initial dye concentrations for both dyes (91.85% for IC and 90.17% for NR). As the initial concentration increases, degradation efficiency decreases. This may be because the number of dye molecules contacting the catalyst surface and the number of dye molecules in the solution rises. This leads to the active sites on the catalyst surface being covered by dye molecules, which interferes with photon arrival and reduces catalytic efficiency. As a result, less OH radical generation occurs on the catalyst surface^23,31^. As a result, increased dye concentration can reduce removal efficiency and reaction rate (Fig. 8).

**Table 6:** Effect of initial dye concentration on adsorption percentage, photocatalytic degradation efficiency, and photocatalytic degradation rate of IC and NR dye.

Initial dye concentration (ppm)	Adsorption percentage (%)		Photocatalytic degradation efficiency		k (min^−1^)		R^2^	
	IC	NR	IC	NR	IC	NR	IC	NR
25	82.92	81.69	91.85	90.17	0.0072	0.00679	0.989	0.924
40	55.25	60.97	67.49	64.37	0.00474	0.00455	0.931	0.957
50	48.9	53.6	49.09	40.28	0.00221	0.00168	0.873	0.9753
75	34.1	30.29	37.41	31.74	0.00043	0.0014	0.97	0.878

#### effect of catalyst loading

An essential practical factor affecting the photocatalytic reaction's effectiveness is catalyst loading. The number of active sites in the reaction system rises due to increased catalyst loading since it increases the concentration of the active material overall. When it comes to the photodegradation removal process, increasing the dose often results in higher removal efficiencies up until a point at which adding more catalyst results in lower light transmittance of the suspension and lower removal efficiencies.

rGO-250 is dissolved in 50 ml of distilled water in a variety of ratios, including 15, 25, 35, 45, and 55 mg. 10 ml of dye solutions with an IC and NR of 25 ppm each and a pH of 10 were combined with 5 ml of this solution. For 30 min, the sample solutions were left in the dark. 3 ml are withheld after 30 min, and UV–vis recordings are made. For roughly 3 h, the remaining sample solution was exposed to sunshine. After every hour, the aliquots were removed, and UV–vis measurements were taken.

**Figure 9:**
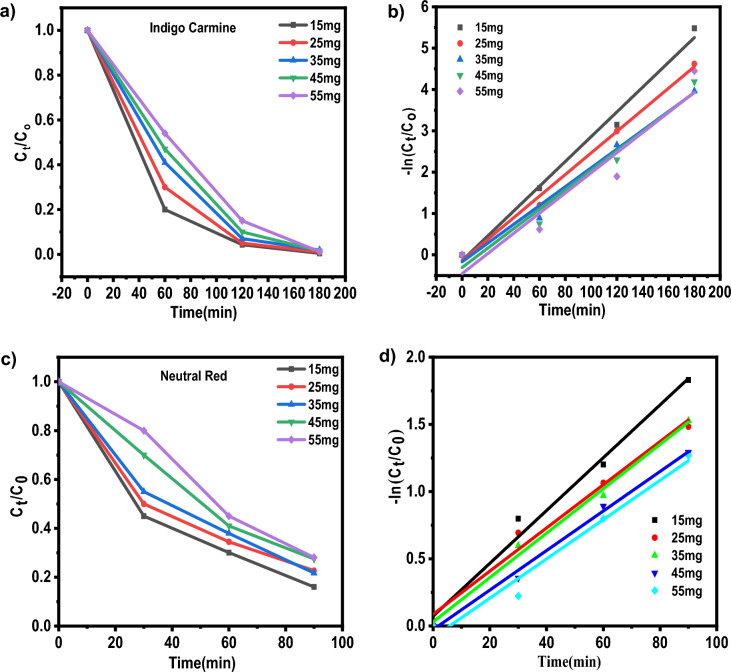
(**a**,**c**) Effect of catalyst loading on photocatalytic degradation of dyes normalized against adsorption. (**c**,**d**) The plot of the log of concentration with time.

As the catalyst loading increased, it was found that the deterioration grew (Table 7). For 15 mg of catalyst, degradation efficiency was ~ 92 for both dyes. The degradation efficiency was determined to be 98.74% for IC and 97.56% for NR, respectively, with a catalyst dosage of 55 mg. Herein, The overall surface area of the photocatalyst grew along with the catalyst concentration. Thus, there were more accessible adsorption sites, which improved the dye adsorption procedure (Fig. 9). Additionally, when the total amount of catalyst particles rose, photon absorption for generating electrons and holes and the rate of the synthesis of reactive oxygen species (ROS) for degrading dye molecules both improved^32,33^.

**Table 7:** Effect of catalyst loading on adsorption percentage, photocatalytic degradation efficiency, and photocatalytic degradation rate of IC and NR dye.

Catalyst loading (mg)	Adsorption percentage (%)		Photocatalytic degradation efficiency		k (min^−1^)		R^2^	
	IC	NR	IC	NR	IC	NR	IC	NR
15	82.92	81.69	91.85	90.17	0.0072	0.00679	0.989	0.924
25	83.45	81.85	95.12	91.59	0.0199	0.019	0.994	0.978
35	86.85	83.18	95.49	93.47	0.0260	0.0165	0.9860	0.992
45	87.39	89.26	98.15	95.29	0.022	0.0147	0.967	0.994
55	91.74	94.81	98.74	97.56	0.023	0.0146	0.918	0.973

Table 8 compares the present work with the existing literature values. The proposed work has the highest degradation efficiency with lower catalyst dosage compared to existing literature for the degradation of IC. In the case of NR dye, it was observed that the rGO catalyst only exhibits a degradation efficiency higher than 90%, whereas the other literature exhibits a degradation efficiency less than 85%. In conclusion, using rGO as a catalyst for both IC and NR degradation demonstrates significant promise in enhancing the efficiency and kinetics of dye removal compared to the existing literature. This innovative approach not only offers an eco-friendly and cost-effective solution for wastewater treatment but also opens up avenues for further research and development in the field of advanced materials and catalysis.

**Table 8:** Comparison of present work with existing literature value.

Catalyst	Dye	pH	Catalyst dosage (g)	Dye concentration (ppm)	Degradation time (minutes)	Degradation efficiency (%)	References
Bi_5_Ti_3_FeO_15_	IC	4	0.06	30	240	97	^34^
Α-Fe_2_O_3_-bentonite	IC	1	0.25	10	90	93	^35^
CaO	IC	9	0.12	500	250	90	^36^
ZnO	IC	7	0.1	90	90	55	^37^
rGO (present work)	**IC**	**10**	***0.055***	**25**	**200**	***98.74***	**–**
Co-mesoporous silica	NR	7	0.075	23	220	81	^38^
[Ni(2-picolinate)·H_2_O]·H_2_O	NR	9	0.03	10	150	82	^39^
Cobalt hexacyanoferrate (II)	NR	5	0.15	17	120	–	^40^
rGO (present work)	**NR**	**10**	***0.055***	**25**	**200**	***97.56***	–

Significant values are in bold and italics.

#### Recyclability

Recyclability studies in photocatalysis are essential for assessing the practicality and sustainability of photocatalytic processes for various applications, such as water purification, air pollution control, and organic pollutant degradation. Recyclability studies aim to determine the ability to reuse the photocatalyst multiple times without significantly losing its catalytic activity. According to the set of photocatalytic tests, rGO-250 is a useful material for the breakdown of both dye molecules. As a result, the ability of this decreased graphene oxide material to degrade dye molecules across consecutive cycles of degradation is tested. The catalyst is removed from each photocatalytic experiment and used for the subsequent cycle of the degradation investigation for this reason. The rGO-250 recovered from each cycle is thoroughly cleaned with deionized water before being added to the catalytic process.

**Figure 10:**
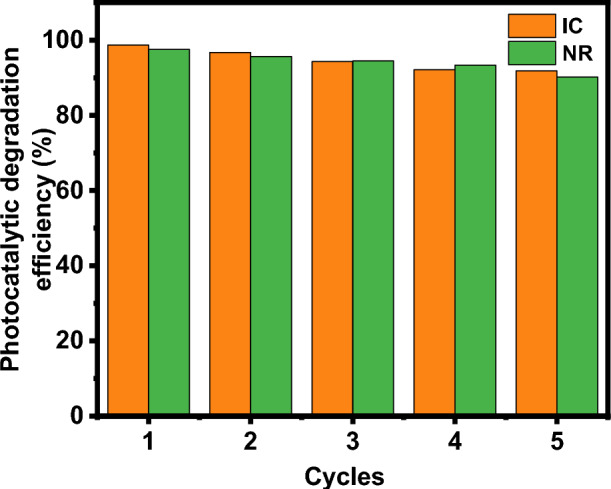
Recyclability analysis of rGO-250 for photodegradation of IC and NR dyes, respectively.

Interestingly, its stability is maintained during the five degradation cycles with barely perceptible alterations. Figure 10 shows the stability of rGO-250 in the photocatalytic dye degradation reaction as a function of cycle number. The percentage degradation was still greater than 90% after five consecutive runs. It attests to the catalyst's structural stability even after five cycles of degradation tests. Under exposure to direct sunshine, it can be employed as a possible catalyst for the degradation of IC and NR dyes^25,28^.

### CO_2_ adsorption performance of rGOs

**Figure 11:**
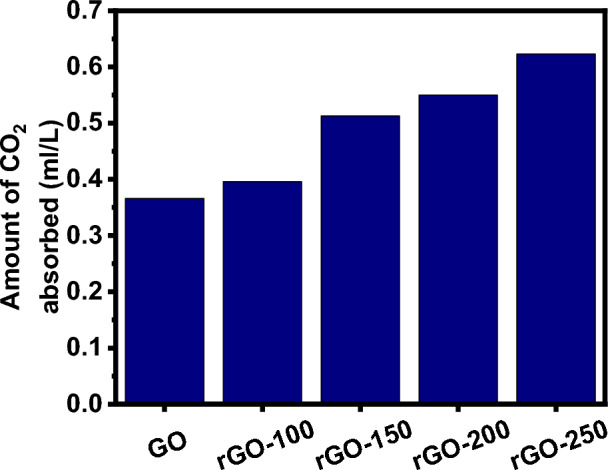
CO_2_ adsorption by GO and rGOs.

Table 9 summarizes the comparison of CO_2_ uptakes by GOs and rGOs. The rGO-250 was the best compared to other adsorbents (Fig. 11). This excellent adsorption performance is attributed to increased surface area at higher reduction temperatures, owing to the significant removal of oxygen functional groups and increased pore volume. After removing surcial oxygen functionalities, graphene's sp^2^- hybridized structure was partially restored at 250 °C^41,42^.

**Table 9:** CO_2_ adsorption by GO and rGOs.

Sl. No	Compound	Amount of CO_2_ adsorbed (ml/L)
1	GO	0.366
2	rGO-100	0.396
3	rGO-150	0.513
4	rGO-200	0.55
5	rGO-250	0.623

## Conclusion

A simple one-pot solvothermal approach was used to fabricate reduced graphene oxide at different annealing temperatures. These rGOs were tested for dye adsorption and CO_2_ adsorption properties. rGO-250 then achieved the best adsorptive removal (29.26% for IC and 25.23% for NR) and photocatalytic degradation (36.28% for IC and 25.73% for NR) of dyes, revealing that the main reasons were increased surface area and narrowing of the band gap. The optimal adsorption (91.74% for IC and 94.81% for NR) and photocatalytic activity (98.74% for IC and 98.56% for NR) of rGO-250 were improved by increasing the amount of catalyst to 55 mg, the initial dye concentration to 25 ppm, and the solution pH to 10. rGO-250 was able to degrade more than 90% of dyes after repeating the reaction up to five times, indicating that it was highly stable and reusable. It was also observed that rGO-250 possesses better CO_2_ adsorption properties than other rGOs.

### Supplementary Information

Supplementary Information.

## Data Availability

The data supporting this study's findings are available at the request of the corresponding author.

## References

[1] Aldalbahi A, El-Naggar ME, El-Newehy MH, Rahaman M, Hatshan MR, Khattab TA (2021). Effects of technical textiles and synthetic nanofibers on environmental pollution. Polymers (Basel).

[2] Kabir SMF, Cueto R, Balamurugan S, Romeo LD, Kuttruff JT, Marx BD, Negulescu II (2019). Removal of acid dyes from textile wastewaters using fish scales by absorption process. Clean Technol..

[3] Lellis B, Fávaro-Polonio CZ, Pamphile JA, Polonio JC (2019). Effects of textile dyes on health and the environment and bioremediation potential of living organisms. Biotechnol. Res. Innov..

[4] Al-Tohamy R, Ali SS, Li F, Okasha KM, Mahmoud YA-G, Elsamahy T, Jiao H, Fu Y, Sun J (2022). A critical review on the treatment of dye-containing wastewater: Ecotoxicological and health concerns of textile dyes and possible remediation approaches for environmental safety. Ecotoxicol. and Environ. Saf..

[5] Dutta S, Gupta B, Kumar Srivastava S, Kumar Gupta A (2021). Recent advances on removing dyes from wastewater using various adsorbents: A critical review. Mater. Adv..

[6] Pavel M, Anastasescu C, State R-N, Vasile A, Papa F, Balint I (2023). Photocatalytic degradation of organic and inorganic pollutants to harmless end products: Assessment of practical application potential for water and air cleaning. Catalysts.

[7] Bi Y, Westerhoff P (2019). High-throughput analysis of photocatalytic reactivity of differing TiO_2_ formulations using 96-well microplate reactors. Chemosphere.

[8] Ahmed A, Singh A, Young S-J, Gupta V, Singh M, Arya S (2023). Synthesis techniques and advances in sensing applications of reduced graphene oxide (rGO) Composites: A review. Compos. Part A Appl. Sci. Manuf..

[9] Melo JF, Junior JHS, Freire TBM, Rigoti E, Pergher SBC, Martínez-Huitle CA, Castro PS (2023). Industrial waste reuse: An alternative source to reduced graphene oxide for preparing electrochemical sensors. Electrochim. Acta.

[10] Mombeshora ET, Muchuweni E (2023). Dynamics of reduced graphene oxide: Synthesis and structural models. RSC Adv..

[11] Majumder P, Gangopadhyay R (2022). Evolution of graphene oxide (GO)-based nanohybrid materials with diverse compositions: An overview. RSC Adv..

[12] Faniyi IO, Fasakin O, Olofinjana B, Adekunle AS, Oluwasusi TV, Eleruja MA, Ajayi EOB (2019). The comparative analyses of reduced graphene oxide (RGO) prepared via green, mild and chemical approaches. SN Appl. Sci..

[13] Wang B, Lan J, Bo C, Gong B, Ou J (2023). Adsorption of heavy metal onto biomass-derived activated carbon: Review. RSC Adv..

[14] Bazan-Wozniak A, Cielecka-Piontek J, Nosal-Wiercińska A, Pietrzak R (2022). Adsorption of organic compounds on adsorbents obtained with the use of microwave heating. Materials (Basel).

[15] Wang J, Wang R, Ma J, Sun Y (2022). Study on the application of shell-activated carbon for the adsorption of dyes and antibiotics. Water.

[16] Kumari M, Pulimi M (2023). Sulfate radical-based degradation of organic pollutants: A review on application of metal–organic frameworks as catalysts. ACS Omega.

[17] Ramesha GK, Ashok V, Muralidhara HB, Sampath S (2011). Graphene and graphene oxide as effective adsorbents toward anionic and cationic dyes. J. Colloid Interface Sci..

[18] Nizam NUM, Hanafiah MM, Mahmoudi E, Mohammad AW, Oyekanmi AA (2022). Effective adsorptive removal of dyes and heavy metal using graphene oxide based Pre-treated with NaOH/H_2_SO_4_ rubber seed shells synthetic graphite Precursor: Equilibrium Isotherm, kinetics and thermodynamic studies. Sep. Purif. Technol..

[19] Ab Aziz NAH, Md Ali UF, Ahmad AA, Mohamed Dzahir MIH, Khamidun MH, Abdullah MF (2023). Non-functionalized oil palm waste-derived reduced graphene oxide for methylene blue removal: Isotherm, kinetics, thermodynamics, and mass transfer mechanism. Arab. J. Chem..

[20] Porcu S, Secci F, Ricci PC (2022). Advances in hybrid composites for photocatalytic applications: A review. Molecules.

[21] Tahir MB, Sohaib M, Sagir M, Rafique M (2022). Role of nanotechnology in photocatalysis. Encycl. Smart Mater..

[22] Sha MS, Devarajan S, Musthafa FN, Eltai NO, Ippili S, Jella V, Yoon S-G, Al-Lohedan H, Ramalingam RJ, Sadasivuni KK (2023). Antibacterial and catalytic performance of rGO-CNT-ZrO_2_ composite. Int. J. Environ. Anal. Chem..

[23] Kumar M, Ansari MNM, Boukhris I, Al-Buriahi MS, Alrowaili ZA, Alfryyan N, Thomas P, Vaish R (2022). Sonophotocatalytic dye degradation using rGO-BiVO_4_ composites. Glob. Chall..

[24] Çeti̇nkaya Gürer S, Kütük N (2021). Effect of reduction temperature and time on the reduction of graphene oxide with white cabbage extract. Bitlis Eren Üniversitesi Fen Bilimleri Dergisi.

[25] Er Siong VL, Mun Lee K, Ching Juan J, Wei Lai C, Hong Tai X, Seong Khe C (2019). Removal of methylene blue dye by solvothermally reduced graphene oxide: A metal-free adsorption and photodegradation method. RSC Adv..

[26] Habte AT, Ayele DW (2019). Synthesis and characterization of reduced graphene oxide (rGO) started from graphene oxide (GO) using the tour method with different parameters. Adv. Mater. Sci. Eng..

[27] Yu-guo Y, Gurunathan S (2017). Combination of graphene oxide–silver nanoparticle nanocomposites and cisplatin enhances apoptosis and autophagy in human cervical cancer cells. Int. J. Nanomed..

[28] Parthipan P, Cheng L, Rajasekar A, Govarthanan M, Subramania A (2021). Biologically reduced graphene oxide as a green and easily available photocatalyst for degradation of organic dyes. Environ. Res..

[29] Zhang S, Fan X, Guan R, Hu Y, Jiang S, Shao X, Wang S, Yue Q (2022). Carbon dots as metal-free photocatalyst for dye degradation with high efficiency within nine minutes in dark. Opt. Mater..

[30] Wang J, Zhu H, Hurren C, Zhao J, Pakdel E, Li Z, Wang X (2015). Degradation of organic dyes by P25-reduced graphene oxide: Influence of inorganic salts and surfactants. J. Environ. Chem. Eng..

[31] Rajendran R, Shrestha LK, Minami K, Subramanian M, Jayavel R, Ariga K (2014). Dimensionally integrated nanoarchitectonics for a novel composite from 0D, 1D, and 2D nanomaterials: RGO/CNT/CeO2 ternary nanocomposites with electrochemical performance. J. Mater. Chem. A.

[32] Sodeinde KO, Olusanya SO, Lawal OS, Sriariyanun M, Adediran AA (2022). Enhanced adsorptional-photocatalytic degradation of chloramphenicol by reduced graphene oxide–zinc oxide nanocomposite. Sci. Rep..

[33] Yu L, Xu W, Liu H, Bao Y (2022). Titanium dioxide–reduced graphene oxide composites for photocatalytic degradation of dyes in water. Catalysts.

[34] Ziyaadini M, Ghashang M (2021). Sunlight-induced photocatalytic degradation of indigo carmine using Bi_5_Ti_3_FeO_15_ layered structure. Optik.

[35] Lubis S, Sitompul DW (2019). Photocatalytic degradation of indigo carmine dye using α-Fe2O3/bentonite nanocomposite prepared by mechanochemical synthesis. IOP Conf. Ser. Mater. Sci. Eng..

[36] Devarahosahalli Veeranna K, Theeta Lakshamaiah M, Thimmasandra Narayan R (2014). Photocatalytic degradation of indigo carmine dye using calcium oxide. Int. J. Photochem..

[37] Taie AA, Dah HM (2017). Photocatalytic degradation of indigo carmine by ZnO photocatalyst under visible light irradiation. Baghdad Sci. J..

[38] Mahfoozi F, Mahmoudi A, Sazegar MR, Nazari K (2021). High-performance photocatalytic degradation of neutral red over cobalt grafted-mesoporous silica under UV irradiation. J. Sol Gel Sci. Technol..

[39] Singh P, Hasija A, Thakur C, Chopra D, Siddiqui KA (2023). Exploring the pH reliant high photocatalytic degradation of organic dyes using H-bonded Ni(II) coordination network. J. Mol. Struct..

[40] Sharma O, Sharma MK (2013). Use of cobalt hexacyanoferrate(II) semiconductor in photocatalytic degradation of neutral red dye. Int. J. ChemTech Res..

[41] Mehra P, Paul A (2022). Decoding carbon-based materials’ properties for high CO_2_ capture and selectivity. ACS Omega.

[42] Politakos N, Cordero-Lanzac T, Tomovska R (2021). Understanding the adsorption capacity for CO_2_ in reduced graphene oxide (rGO) and modified ones with different heteroatoms in relation to surface and textural characteristics. Appl. Sci..

