# Supplementary material for: Photocatalytic degradation of organic dyes using reduced graphene oxide (rGO)
Source: Sci Rep. 2024 Feb 13;14:3608. doi: 10.1038/s41598-024-53626-8 (PMC10864344; doi:10.1038/s41598-024-53626-8)
Supplement: Supplementary file 1 — Supplementary Information. [file 41598_2024_53626_MOESM1_ESM.docx]

**Figure S1.** UV-Vis analysis of NR alone.

**Figure S2.** UV-Vis analysis of NR dye with rGOs.

**Figure S3.** UV-Vis analysis of IC alone.

**Figure S4.** UV-Vis analysis of IC dye with rGOs.

**Table S1.** Statistical analysis of pH effect of NR dye.

| **pH of Dye** | | **DF** | **SS** | **MS** | **F value** | **P>F** |
| --- | --- | --- | --- | --- | --- | --- |
| **pH3** | **Model** | 1 | 0.02848 | 0.02848 | 12.20623 | 0.02504 |
|  | **Error** | 4 | 0.00933 | 0.00233 |  |  |
|  | **Total** | 5 | 0.03781 |  |  |  |
| **pH7** | **Model** | 1 | 0.07933 | 0.07933 | 28.92575 | 0.00578 |
|  | **Error** | 4 | 0.01097 | 0.00274 |  |  |
|  | **Total** | 5 | 0.0903 |  |  |  |
| **pH10** | **Model** | 1 | 0.25513 | 0.25513 | 13.19492 | 0.02211 |
|  | **Error** | 4 | 0.07734 | 0.01934 |  |  |
|  | **Total** | 5 | 0.33247 |  |  |  |

**Table S2.** Statistical analysis of initial dye concentration of IC dye.

| **Initial Dye concentration** | | **DF** | **SS** | **MS** | **F value** | **P>F** |
| --- | --- | --- | --- | --- | --- | --- |
| **25 ppm** | **Model** | 1 | 0.78737 | 0.78737 | 44.97834 | 8.78753E-5 |
|  | **Error** | 9 | 0.15755 | 0.01751 |  |  |
|  | **Total** | 10 | 0.94492 |  |  |  |
| **40 ppm** | **Model** | 1 | 0.20015 | 0.20015 | 16.03135 | 0.00309 |
|  | **Error** | 9 | 0.11237 | 0.01249 |  |  |
|  | **Total** | 10 | 0.31252 |  |  |  |
| **50 ppm** | **Model** | 1 | 0.00826 | 0.00826 | 294.76293 | 3.47373E-8 |
|  | **Error** | 9 | 2.52132E-4 | 2.80146E-5 |  |  |
|  | **Total** | 10 | 0.00851 |  |  |  |
| **75 ppm** | **Model** | 1 | 0.01528 | 0.01528 | 179.65825 | 2.98527E-7 |
|  | **Error** | 9 | 7.65246E-4 | 8.50273E-5 |  |  |
|  | **Total** | 10 | 0.01604 |  |  |  |

**Table S3.** Statistical analysis of initial dye concentration of NR dye.

| **Initial Dye concentration** | | **DF** | **SS** | **MS** | **F value** | **P>F** |
| --- | --- | --- | --- | --- | --- | --- |
| **25 ppm** | **Model** | 1 | 0.92433 | 0.92433 | 54.61655 | 7.6906E-5 |
|  | **Error** | 8 | 0.13539 | 0.01692 |  |  |
|  | **Total** | 9 | 1.05972 |  |  |  |
| **40 ppm** | **Model** | 1 | 0.09269 | 0.09269 | 180.29868 | 9.06455E-7 |
|  | **Error** | 8 | 0.00411 | 5.14099E-4 |  |  |
|  | **Total** | 9 | 0.0968 |  |  |  |
| **50 ppm** | **Model** | 1 | 0.06484 | 0.06484 | 316.39383 | 1.02154E-7 |
|  | **Error** | 8 | 0.00164 | 2.04925E-4 |  |  |
|  | **Total** | 9 | 0.06648 |  |  |  |
| **75 ppm** | **Model** | 1 | 0.02112 | 0.02112 | 57.59141 | 6.37092E-5 |
|  | **Error** | 8 | 0.00293 | 3.6666E-4 |  |  |
|  | **Total** | 9 | 0.02405 |  |  |  |

**Table S4.** Statistical analysis of the effect of catalyst loading on IC dye.

| **Effect of catalyst loading** | | **DF** | **SS** | **MS** | **F value** | **P>F** |
| --- | --- | --- | --- | --- | --- | --- |
| **15 mg** | **Model** | 1 | 16.15797 | 16.15797 | 191.55653 | 0.00418 |
|  | **Error** | 2 | 0.1687 | 0.08435 |  |  |
|  | **Total** | 3 | 16.32667 |  |  |  |
| **25 mg** | **Model** | 1 | 12.25021 | 12.25021 | 336.9031 | 0.00296 |
|  | **Error** | 2 | 0.07272 | 0.03636 |  |  |
|  | **Total** | 3 | 12.32293 |  |  |  |
| **35 mg** | **Model** | 1 | 9.31575 | 9.31575 | 141.06407 | 0.00701 |
|  | **Error** | 2 | 0.13208 | 0.06604 |  |  |
|  | **Total** | 3 | 9.44783 |  |  |  |
| **45 mg** | **Model** | 1 | 9.96424 | 9.96424 | 60.26212 | 0.01619 |
|  | **Error** | 2 | 0.3307 | 0.16535 |  |  |
|  | **Total** | 3 | 10.29494 |  |  |  |
| **55 mg** | **Model** | 1 | 10.69516 | 10.69516 | 22.41599 | 0.04183 |
|  | **Error** | 2 | 0.95424 | 0.47712 |  |  |
|  | **Total** | 3 | 11.64941 |  |  |  |

**Table S5.** Statistical analysis of the effect of catalyst loading on NR dye.

| **Effect of catalyst loading** | | **DF** | **SS** | **MS** | **F value** | **P>F** |
| --- | --- | --- | --- | --- | --- | --- |
| **15 mg** | **Model** | 1 | 1.7365 | 1.7365 | 131.00917 | 0.00755 |
|  | **Error** | 2 | 0.02651 | 0.01325 |  |  |
|  | **Total** | 3 | 1.76301 |  |  |  |
| **25 mg** | **Model** | 1 | 1.16154 | 1.16154 | 90.41653 | 0.01088 |
|  | **Error** | 2 | 0.02569 | 0.01285 |  |  |
|  | **Total** | 3 | 1.18724 |  |  |  |
| **35 mg** | **Model** | 1 | 1.22603 | 1.22603 | 275.52983 | 0.00361 |
|  | **Error** | 2 | 0.0089 | 0.00445 |  |  |
|  | **Total** | 3 | 1.23493 |  |  |  |
| **45 mg** | **Model** | 1 | 0.97286 | 0.97286 | 362.40855 | 0.00275 |
|  | **Error** | 2 | 0.00537 | 0.00268 |  |  |
|  | **Total** | 3 | 0.97823 |  |  |  |
| **55 mg** | **Model** | 1 | 0.95989 | 0.95989 | 74.64013 | 0.01313 |
|  | **Error** | 2 | 0.02572 | 0.01286 |  |  |
|  | **Total** | 3 | 0.98561 |  |  |  |
